# Supplementary material for: Whole genome sequencing and imputation in isolated populations identify genetic associations with medically-relevant complex traits
Source: Nat Commun. 2017 May 26;8:15606. doi: 10.1038/ncomms15606 (PMC5458552; doi:10.1038/ncomms15606)
Supplement: Supplementary Information — Supplementary Figures, Supplementary Tables, Supplementary Notes, Supplementary Methods and Supplementary References [file ncomms15606-s1.pdf]

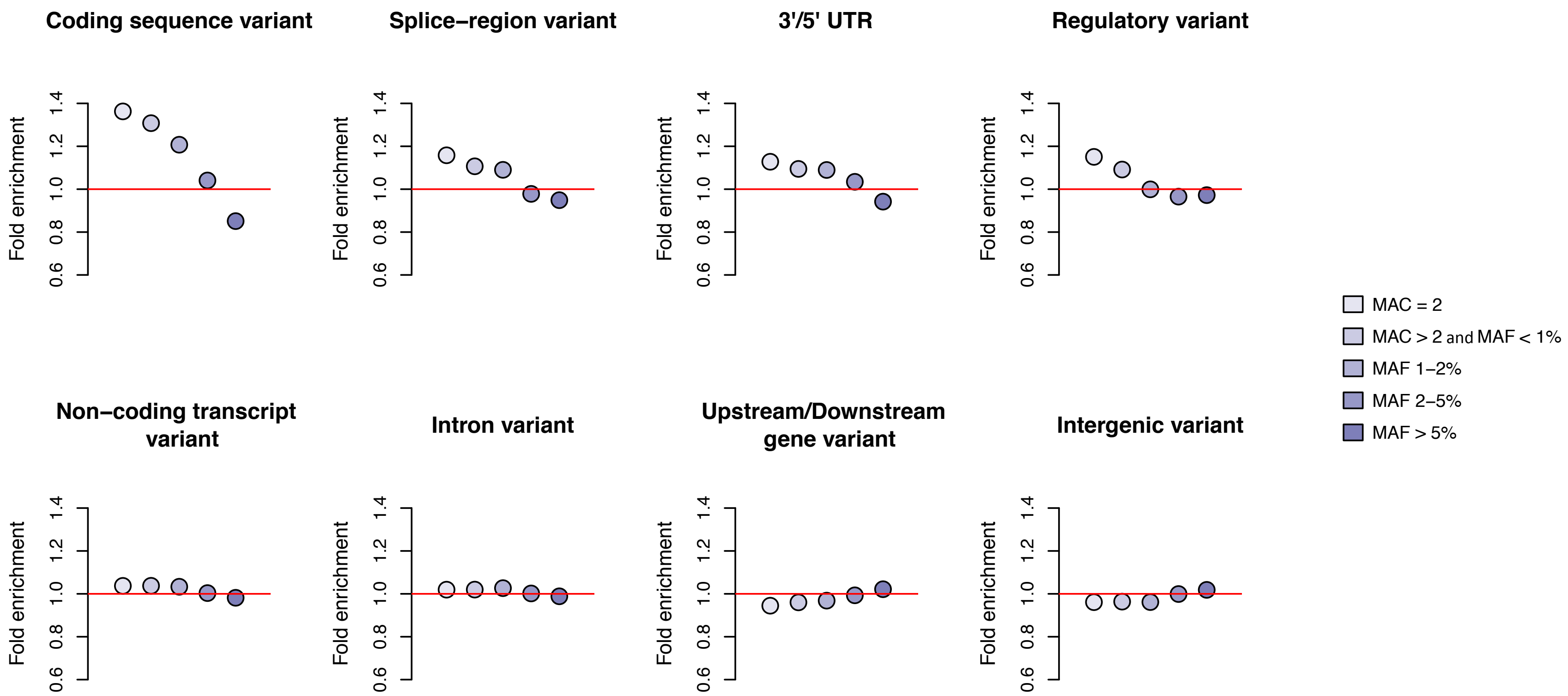

Supplementary Figure 1: MANOLIS WGS variant consequences and allele frequencies.

MAF, minor allele frequencies; MAC, minor allele count. The x-axis shows MAF category, the y axis shows fold enrichment. Enrichment is calculated by dividing the proportion of variants of a given consequence observed in a MAF bin by the proportion of variants of the same consequence in the total dataset. For numerical values see Supplementary Table 3.

a.

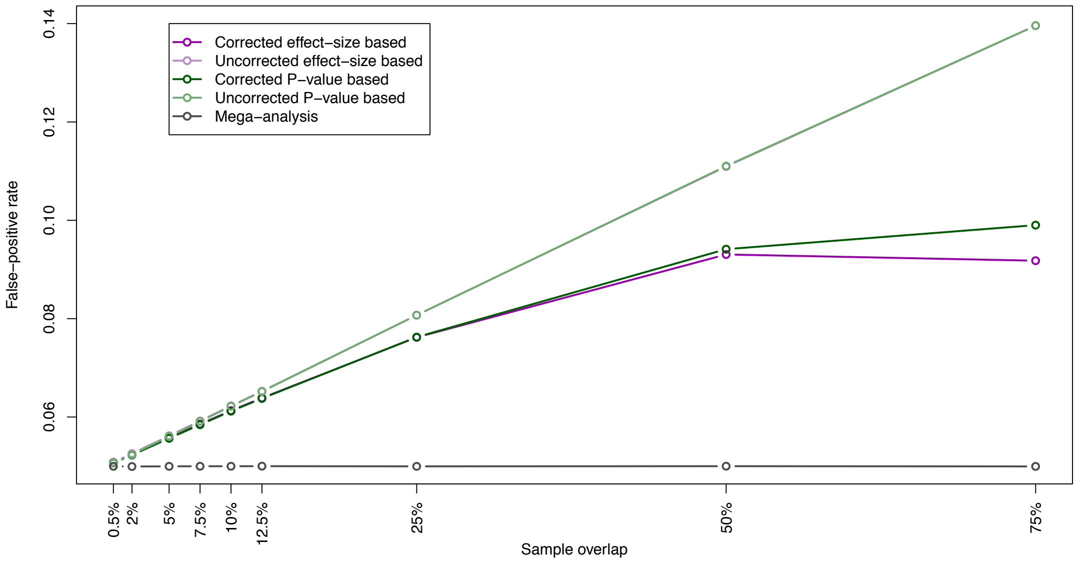

b.

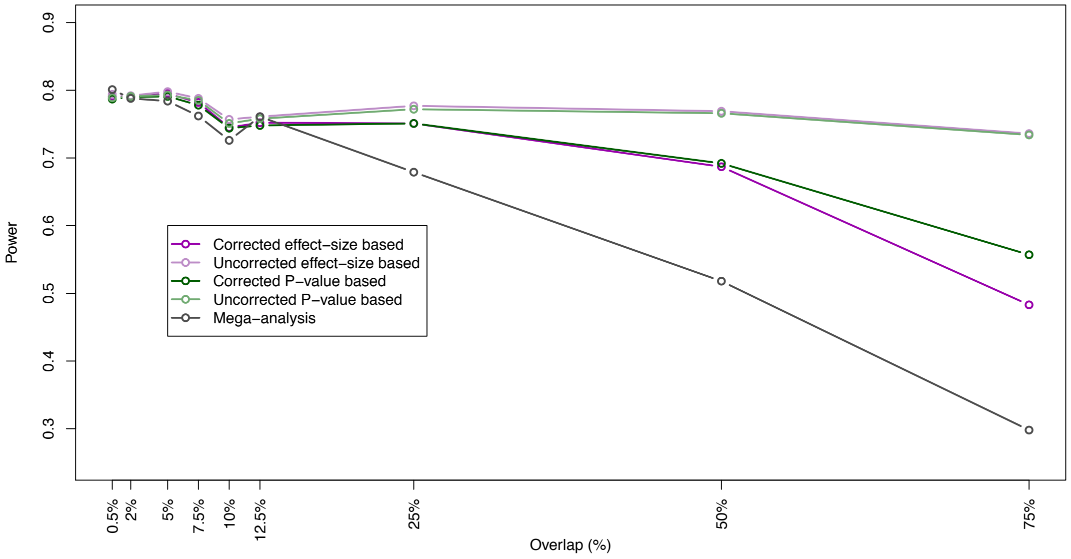

Supplementary Figure 2: Power and false-positive rate for METACARPA under extensive sample overlap.

a. Empirical false-positive rate as a function of sample overlap in 1,000 repeats of a meta-analysis of two studies including 2,000 samples each, at a significance threshold of 5.00×10^{-8}. **b.** Empirical power of the four tests implemented in METACARPA as a function of sample overlap in the same simulation setting. Power is calculated as the discovery rate of a SNP explaining 1% of a standard normal phenotype under the same simulation scenario (e.g. a MAF of 1% and an effect size of 0.705, or a MAF of 20% and an effect size of 0.176).

METACARPA
lambda=0.9901

GWAMA
lambda=0.9999

mega-analysis
lambda=0.9746

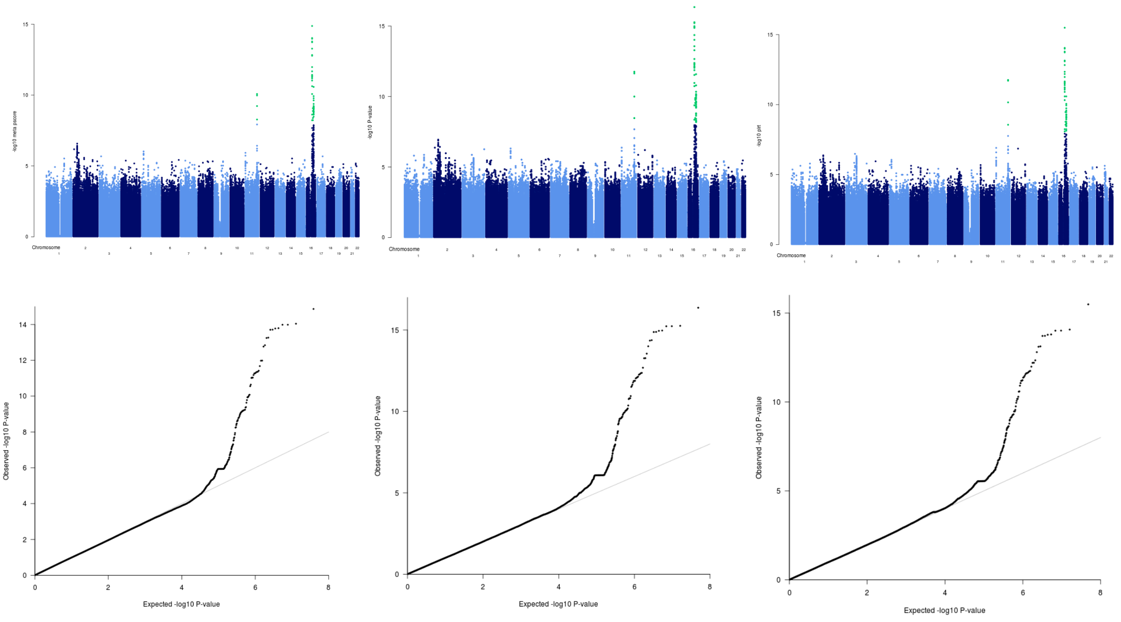

Supplementary Figure 3: Comparison between three meta-analysis strategies on the HELIC MANOLIS dataset.

The trait being meta-analysed is HDL. The top 3 panels are the Manhattan plots for the 3 analysis approaches taken, with the corresponding qq-plots in the lower panels.

a. Association between rs140087759 and WHR in HELIC MANOLIS.

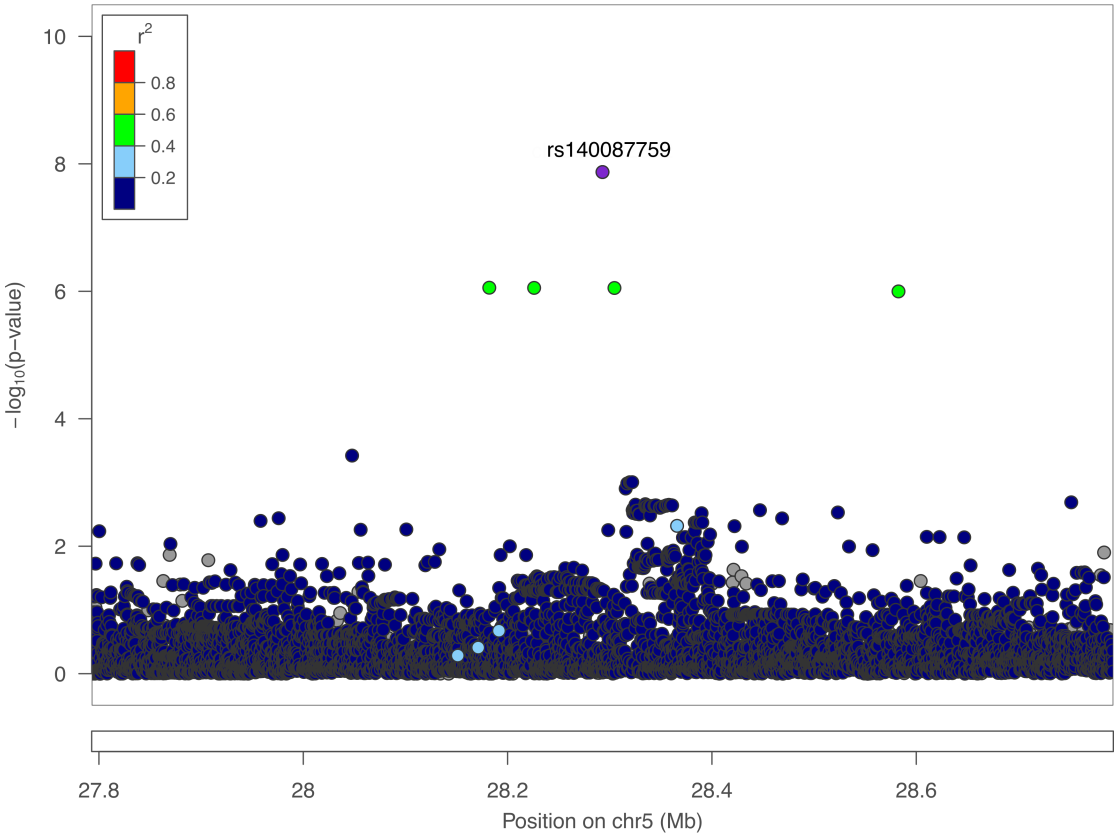

b. Association between rs13382259 and DBP in HELIC Pomak.

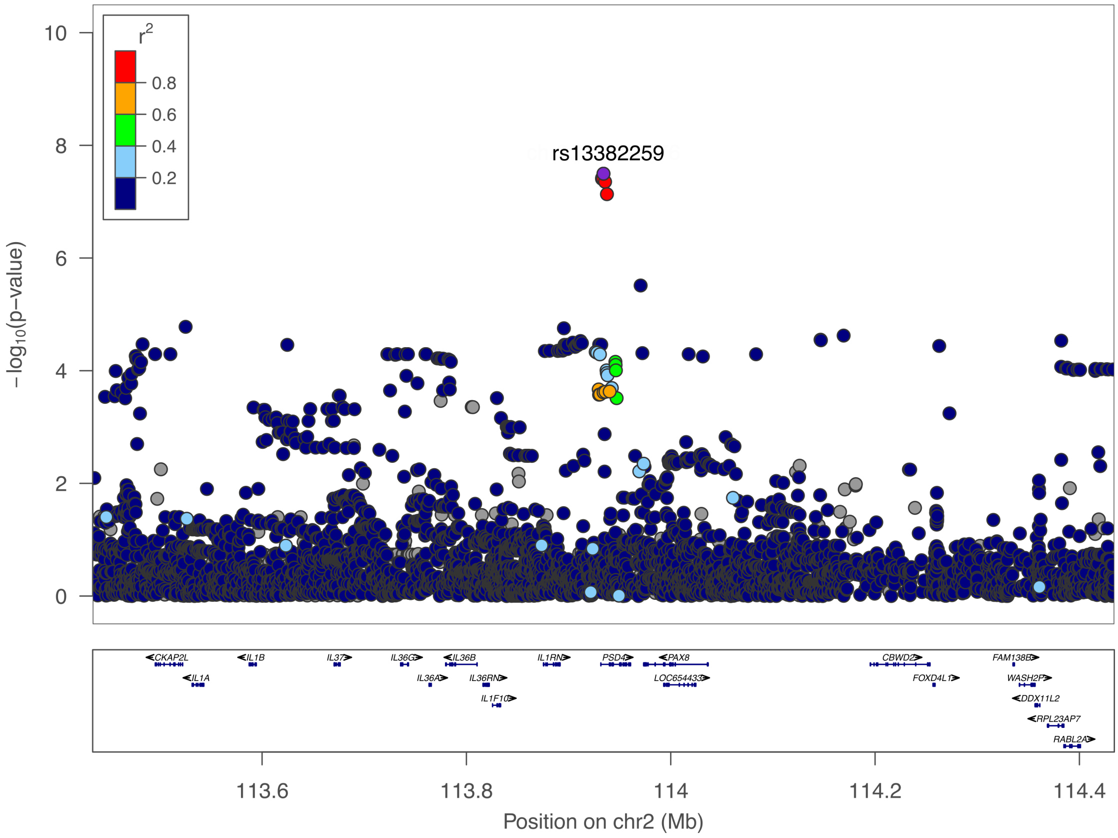

c. Association between rs6131100 and FGadjBMI in HELIC Pomak.

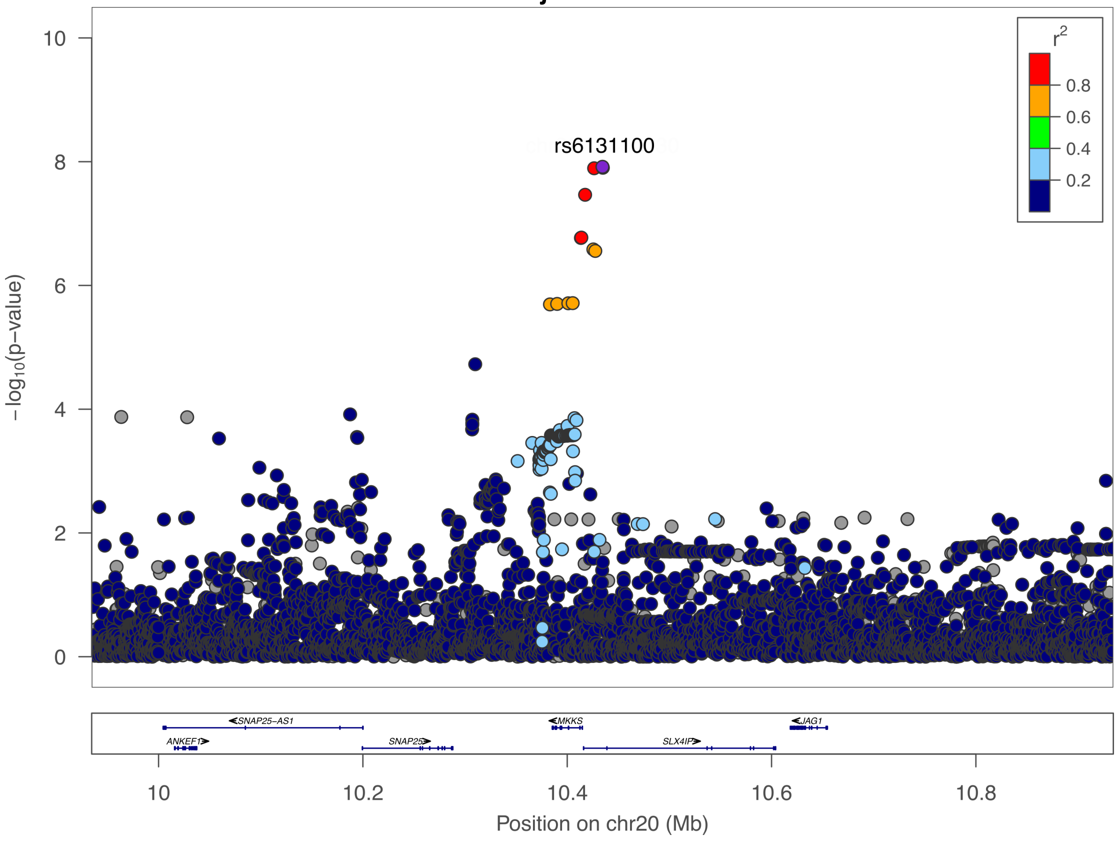

d. Association between rs79748197 and WBC in HELIC Pomak.

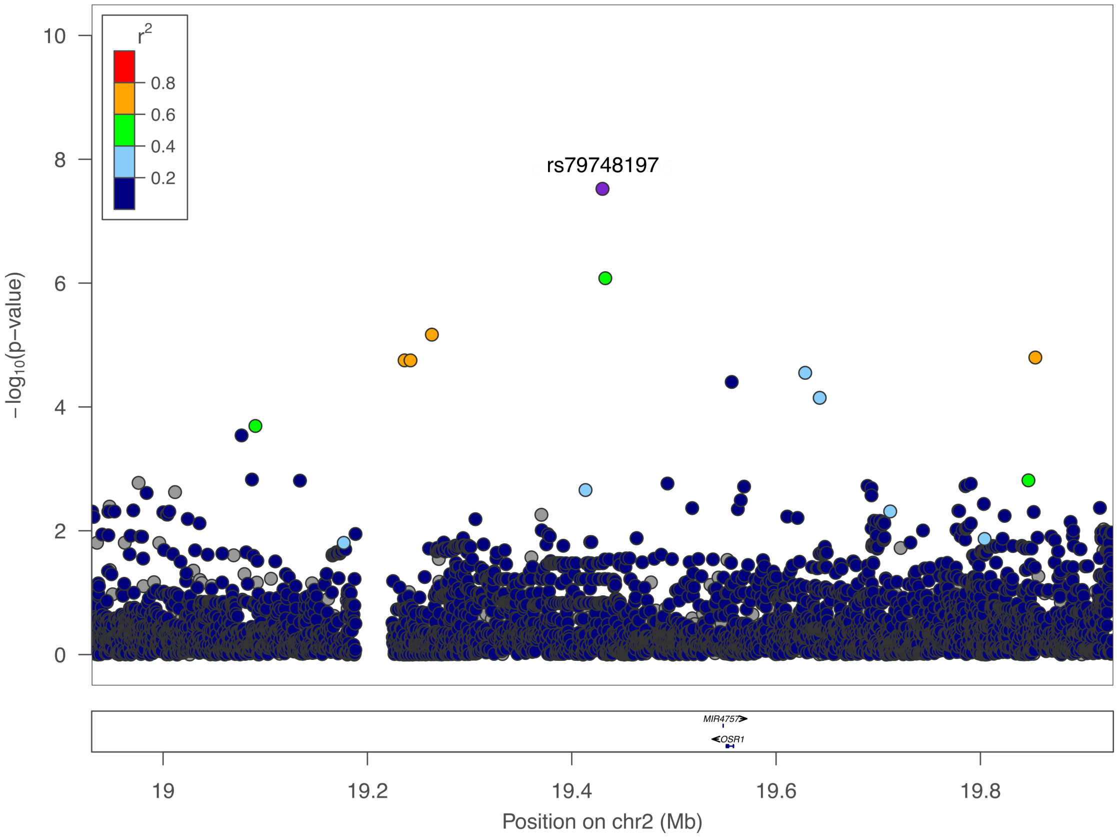

e. Association between rs557129696 and HGB in HELIC Pomak.

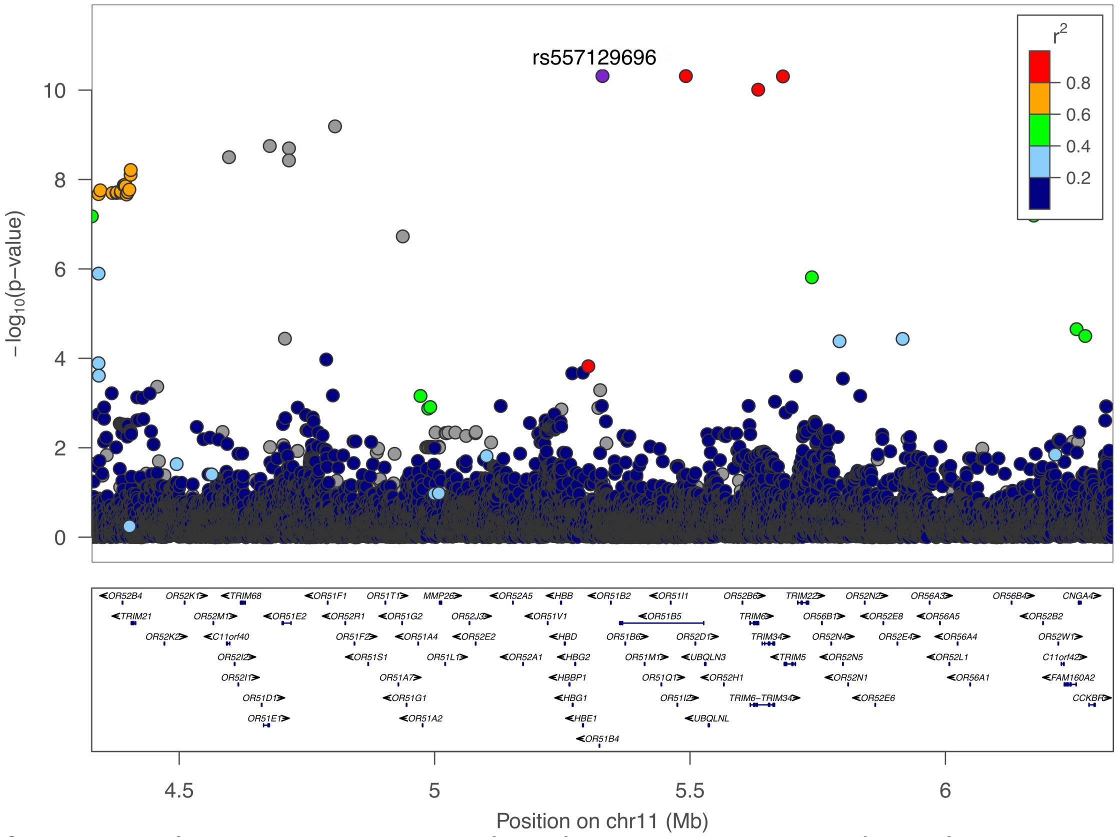

f. Association between rs112037309 and weight in HELIC MANOLIS and Pomak.

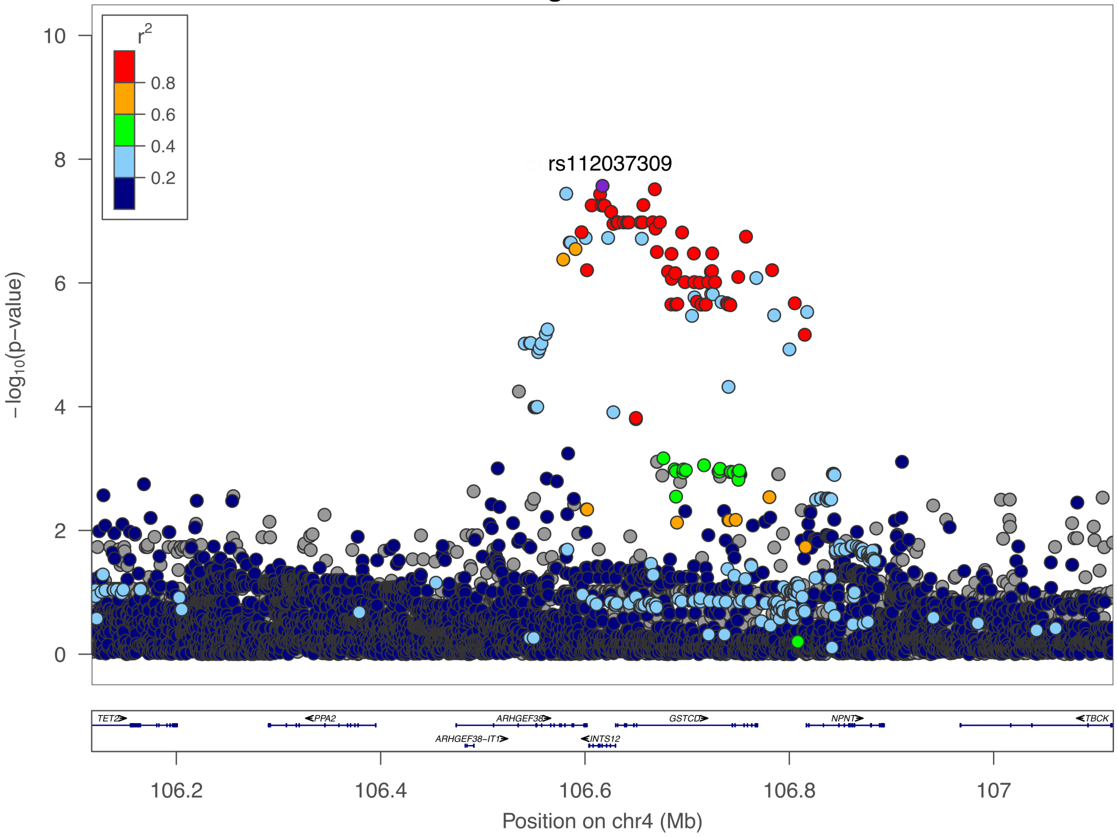

Supplementary Figure 4: Regional association plots for the novel signals.
LocusZoom was used to create the plots (http://csg.sph.umich.edu/locuszoom/). The LD was generated empirically using the OmniExome imputed genotypes from the appropriate cohort. For the MANOLIS and Pomak meta-analysis the Pomak OmniExome data was used for the LD calculation. The

r^2 correlation is represented by different colours (grey, no info; dark blue, 0–0.2; light blue, 0.2–0.4; green, 0.4–0.6; yellow, 0.6–0.8; red, 0.8–1.0). $-\log_{10}$(p-value) is the Wald test *P* value from the association analysis using METACARPA.

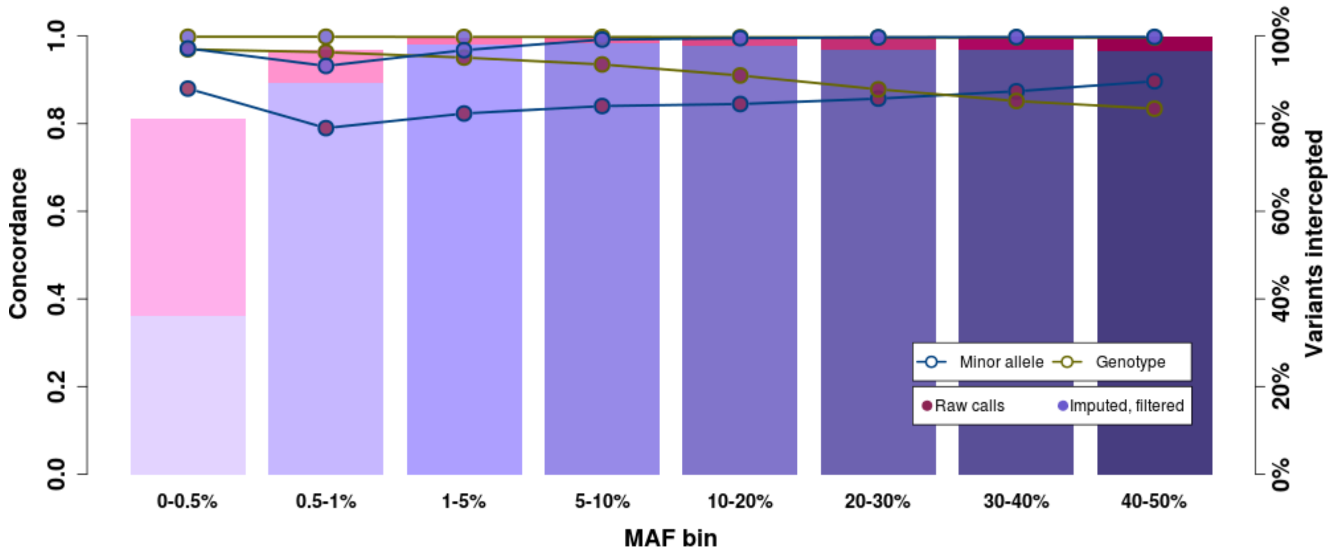

Supplementary Figure 5: Variants called, genotype and minor allele concordance pre- and post-filtering and imputation in the 4x WGS data compared to the array data.

Bars indicate the proportion of variants present in the chip data that were called in the WGS data, per given MAF category. Pink-red bars represent raw sequencing variants (pre-filtering), purple bars represent filtered variants following VQSR, sample and variant-level QC, and phasing (post-filtering). The blue curves indicate minor allele concordance, the grey ones genotype concordance. Points for concordance are coloured in pink-red for the unfiltered dataset and purple for the filtered dataset. The y-axis on the right (Variants Intercepted) refers to the bars and the y-axis on the left (Concordance) refers to the curves.

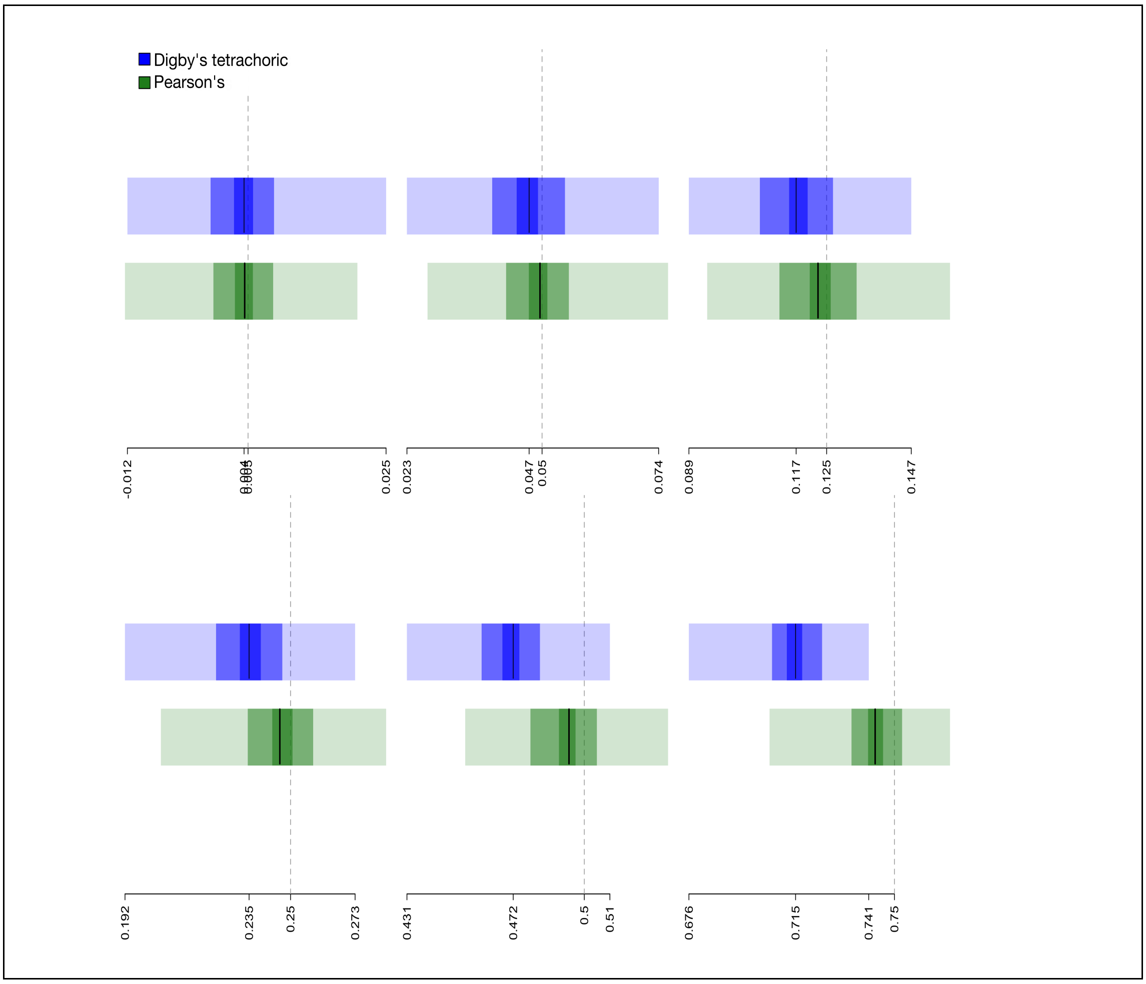

Supplementary Figure 6: Accuracy of tetrachoric correlation of z-scores under different trait architectures. Accuracy when all SNPs are under the null, for increasing sample overlap. Green boxes represent the quintiles of the correlation estimates for Pearson's correlation of z-scores, blue boxes the quintiles of Digby's estimator of tetrachoric correlation. Real expected correlation is represented by the dashed grey line.

**Genomic region**	**Variant count (% of total)**	**Mean SNV density (kbp^{-1})**	**SEM**
Splice region	11,232 (0.12%)	1.924	0.018
Coding	73,310 (0.76%)	2.096	0.016
UTR	133,097 (1.37%)	2.620	0.024
Intronic	4,878,515 (50.27%)	3.339	0.049
Upstream or Downstream	937,271 (9.66%)	3.494	0.010
Intergenic	3,670,836 (37.83%)	3.657	0.016
Whole genome	9,554,503	3.570	-

Supplementary Table 1: HELIC MANOLIS annotation summary.

Average densities of single nucleotide variants by functional annotation.

MAF category	3'/5' UTR	Coding sequence variant	Intergenic variant	Intron variant	Non-coding transcript variant	Splice-region variant	Regulatory region variant	Upstream/ Downstream gene variant	MAF-bin total
**MAC = 2**	10,506 (1.128)	8,079 (1.363)	269,160 (0.961)	393,877 (1.020)	12,176 (1.037)	1,077 (1.158)	23,053 (1.151)	62,351 (0.945)	780,279
**MAC > 2 and MAF <= 1%**	11,659 (1.095)	8,868 (1.308)	308,623 (0.964)	450,591 (1.020)	13,929 (1.038)	1,177 (1.107)	25,012 (1.092)	72,480 (0.960)	892,339
**MAF 1-2%**	13,403 (1.090)	9,453 (1.207)	355,599 (0.962)	523,814 (1.027)	16,016 (1.033)	1,339 (1.090)	26,438 (0.999)	84,401 (0.968)	1,030,463
**MAF 2-5%**	16,173 (1.034)	10,357 (1.041)	469,698 (0.999)	649,806 (1.002)	19,777 (1.004)	1,527 (0.978)	32,465 (0.965)	110,040 (0.993)	1,309,843
**MAF > 5%**	62,307 (0.942)	35,836 (0.851)	2,025,615 (1.019)	2,712,522 (0.989)	81,837 (0.982)	6,267 (0.949)	138,347 (0.972)	478,848 (1.022)	5,541,579
**Consequence total**	114,048	72,593	3,428,695	4,730,610	143,735	11,387	245,315	808,120	9,554,503

Supplementary Table 2: MANOLIS reference panel variant consequence and allele frequency summary.

Numbers in parentheses indicate the fold enrichment of a consequence in the corresponding MAF bin comparing to the frequency of that consequence in the total dataset.

AF category	MANOLIS + UK10K	MANOLIS + 1000Genomes	MANOLIS + UK10K + 1000Genomes	Unique in MANOLIS	Unique (%)	Distribution of unique variants*
MAC = 2	94,906	84,099	351,662	**249,612**	31.99%	47.59%
MAC > 2 & MAF <= 1%	84,004	103,054	531,176	**174,105**	19.51%	33.19%
MAF > 1% & MAF <= 2%	47,665	100,075	804,614	**78,109**	7.58%	14.89%
MAF > 2% & MAF <= 5%	12,256	91,177	1,187,117	**19,293**	1.47%	3.68%
MAF > 5%	2,633	388,369	5,147,197	**3,380**	0.06%	0.64%
Total:	241,464	766,774	8,021,766	**524,499**	5.49%	100%

Supplementary Table 3: HELIC MANOLIS reference panel summary.

Comparison of the HELIC MANOLIS reference panel variants with the UK10K and 1000 Genomes Project reference panel variants. * Indicates how the number of unique variants are distributed across MAF categories.

Consequence	MAF bin	Shared			Unique to HELIC MANOLIS			Enrichment	*P*
		Variant count	Variant count in MAF bin	Proportion	Variant count	Variant count in MAF bin	Proportion		
Regulatory variant	MAC=2	14,062	530,667	0.026	8,991	249,612	0.036	1.3593	**3.64×10^{-117}**
	≤1%	18,599	718,234	0.026	6,413	174,105	0.037	1.4224	**2.84×10^{-134}**
	≤5%	55,520	2,242,904	0.025	3,383	97,402	0.035	1.4031	**8.71×10^{-83}**
	>5%	138,243	5,538,199	0.025	104	3,380	0.031	1.2327	1.00×10^{0}
UTR	MAC=2	7,070	530,667	0.013	3,436	249,612	0.014	1.0332	1.00×10^{0}
	≤1%	9,148	718,234	0.013	2,511	174,105	0.014	1.1323	**9.42×10^{-7}**
	≤5%	28,232	2,242,904	0.013	1,344	97,402	0.014	1.0962	**3.11×10^{-2}**
	>5%	62,265	5,538,199	0.011	42	3,380	0.012	1.1052	1.00×10^{0}
Coding variant	MAC=2	5,037	530,667	0.009	3,042	249,612	0.012	1.2839	**1.95×10^{-26}**
	≤1%	6,750	718,234	0.009	2,118	174,105	0.012	1.2944	**5.84×10^{-24}**
	≤5%	18,665	2,242,904	0.008	1,145	97,402	0.012	1.4126	**9.14×10^{-29}**
	>5%	35,826	5,538,199	0.006	10	3,380	0.003	0.4574	4.73×10^{-1}
Non-coding transcript variant	MAC=2	8,193	530,667	0.015	3,983	249,612	0.016	1.0335	1.00×10^{0}
	≤1%	11,076	718,234	0.015	2,853	174,105	0.016	1.0626	1.18×10^{-1}
	≤5%	34,268	2,242,904	0.015	1,525	97,402	0.016	1.0248	1.00×10^{0}
	>5%	81,785	5,538,199	0.015	52	3,380	0.015	1.0418	1.00×10^{0}
Splice variant	MAC=2	706	530,667	0.001	371	249,612	0.001	1.1172	1.00×10^{0}
	≤1%	942	718,234	0.001	235	174,105	0.001	1.0291	1.00×10^{0}
	≤5%	2,734	2,242,904	0.001	132	97,402	0.001	1.1118	1.00×10^{0}
	>5%	6,266	5,538,199	0.001	1	3,380	0	0.2615	1.00×10^{0}
Intergenic variant	MAC=2	185,391	530,667	0.349	83,769	249,612	0.336	0.9606	2.90×10^{-31}
	≤1%	250,747	718,234	0.349	57,876	174,105	0.332	0.9522	6.44×10^{-38}
	≤5%	792,496	2,242,904	0.353	32,801	97,402	0.337	0.9531	9.94×10^{-25}
	>5%	2,024,326	5,538,199	0.366	1,289	3,380	0.381	1.0433	1.00×10^{0}
Up/down stream	MAC=2	43,485	530,667	0.082	18,866	249,612	0.076	0.9224	1.37×10^{-20}
	≤1%	58,941	718,234	0.082	13,539	174,105	0.078	0.9476	1.25×10^{-7}
	≤5%	187,100	2,242,904	0.083	7,341	97,402	0.075	0.9035	1.70×10^{-17}
	>5%	478,598	5,538,199	0.086	250	3,380	0.074	0.8559	3.49×10^{-1}
Intron variant	MAC=2	266,723	530,667	0.503	127,154	249,612	0.509	1.0135	**7.20×10^{-7}**
	≤1%	362,031	718,234	0.504	88,560	174,105	0.509	1.0091	**1.84×10^{-2}**
	≤5%	1,123,889	2,242,904	0.501	49,731	97,402	0.511	1.0189	**2.19×10^{-7}**
	>5%	2,710,890	5,538,199	0.489	1,632	3,380	0.483	0.9864	1.00×10^{0}

Supplementary Table 4: Distribution of the consequences for the variants exclusive to the MANOLIS reference panel compared to those shared with UK10K and/or 1000 Genomes.

Consequence: each variant was assigned a consequence according to Ensembl, then consequence terms were pooled into one of the eight categories (see methods for details); MAF, minor allele frequency; MAC, minor allele count; Shared, variants present in MANOLIS and also UK10K and/or 1000 Genomes; Variant count, number of variants in the given consequence category; Variant count in MAF bin, total number of variants in the MAF bin. Proportion: Variant count/ Variant count in MAF bin. Enrichment, proportion variants unique to MANOLIS/proportion of variants shared; *P*, *P* from the Bonferroni corrected two-sided proportions test; Bold indicates significant enrichment (*P*<0.05), underlined *P* indicates significant depletion (*P*<0.05)

Trait	Cohorts	Variant	Genotype comparison						Directly typed genotype association							
			MANOLIS			Pomak										
			n	PPV	Concordance	n	PPV	Concordance	EA	NEA	EAF	Effects	beta	se	*P*	N
**Novel Signals**																
HDL	MANOLIS	Chr16:70790626	1316	0.947	0.947	1442	NA	NA	T	C	0.007	--	-1.380	0.237	6.11×10^{-9}	1306
TG	MANOLIS	rs145556679	1315	1	0.871	1441	NA	0	C	G	0.010	--	-1.266	0.199	2.22×10^{-10}	1302
VLDL									C	G	0.010	--	-1.272	0.200	1.83×10^{-10}	1304
WHR	MANOLIS	rs140087759	1314	0.960	0.828	1440	0.958	1	T	C	0.009	++	1.198	0.228	1.51×10^{-7}	1110
DBP	Pomak	rs13382259	1315	1	1	1440	1	1	T	A	0.043	++	0.678	0.108	3.69×10^{-10}	1017
FGBMIadj	Pomak	rs6131100	1304	1	1	1441	1	1	A	T	0.037	--	-0.910	0.149	1.08×10^{-9}	628
WBC	Pomak	rs79748197	1313	0.667	0.952	1440	1	1	G	A	0.007	--	-1.133	0.235	1.36×10^{-6}	1384
HGB	Pomak	rs557129696	1315	1	1	1441	1	1	G	T	0.004	--	-1.981	0.310	1.67×10^{-10}	1374
Weight*	MANOLIS and Pomak	rs17262443	1287	1	1	1440	1	1	C	T	0.074	++++	0.273	0.056	1.20×10^{-6}	2465
**Known Signals**																
LDL	MANOLIS and Pomak	rs7412	Directly typed			Directly typed										
TC																
CRP	MANOLIS and Pomak	rs7553007	Directly typed			Directly typed										
VLDL	MANOLIS and Pomak	rs964184	Directly typed			Directly typed										
TG																
TG	MANOLIS	rs76353203	Directly typed			Directly typed										
HDL																
LDL	MANOLIS and Pomak	rs150641967	1318	0.839	1	1450	1	1	T	G	0.078	----	-0.288	0.052	2.16×10^{-8}	2724
TC									T	G	0.078	----	-0.280	0.052	6.24×10^{-8}	2724
TG									T	G	0.077	----	-0.272	0.052	1.63×10^{-7}	2720
VLDL									T	G	0.078	----	-0.273	0.052	1.55×10^{-7}	2725
HDL	MANOLIS and Pomak	rs35237252	1315	1	0.999	1429	0.9961	1	A	C	0.274	++++	0.177	0.031	1.00×10^{-8}	2731
HDL	MANOLIS and Pomak	rs200751500	1284	0.823	0.993	1425	0.8930	0.997	A	C	0.386	++++	0.265	0.028	5.69×10^{-21}	2730
MCH	MANOLIS and Pomak	rs1331309	1306	0.998	1	1439	0.9985	1	G	T	0.223	++++	0.192	0.036	9.83×10^{-8}	2439
WBC	Pomak	rs9804550	1316	0.946	1	1439	1	1	T	C	0.054	++	0.480	0.086	2.14×10^{-8}	1383
MCH									T	C	0.056	--	-0.595	0.084	$1.62 \times 10-^{12}$	1360
MCHC									T	C	0.056	++	0.922	0.080	8.39×10^{-31}	1380
MCV									T	C	0.055	--	-1.029	0.081	3.84×10^{-37}	1372
RBC									T	C	0.056	++	0.461	0.082	1.78×10^{-8}	1422

Supplementary Table 5: Validation results. Directly-genotyped genotype concordance and association results for the novel and known signals. Cohorts, cohorts in which the signal arose; n, the number of samples with non-missing imputed and directly-typed genotypes; PPV, minor allele positive predictive value; Concordance, minor allele concordance; EA, effect allele; NEA, non-effect allele; EAF, effect allele frequency; Effects, the direction of effect for each of the studies included in the analysis; *P*, Wald test *P* value from the association analysis using METACARPA; N, the number of samples in the analysis. NA, indicates that the samples were monomorphic; Directly typed, these variants were directly typed and the intensity clustering was good; TG, triglycerides; VLDL, very low density lipoprotein cholesterol; HDL, high-density lipoprotein cholesterol; DBP, diastolic blood pressure; WHR, waist-to-hip ratio; FGBMIadjusted, fasting glucose adjusted for body mass index; WBC, white blood cells; HGB, haemoglobin; LDL, low-density lipoprotein cholesterol; TC, total cholesterol; CRP, C-reactive protein; MCH, mean corpuscular haemoglobin; MCHC, mean corpuscular haemoglobin concentration; MCV, mean corpuscular volume; RBC, red blood cells. *Proxy validated for rs112037309 (r^2=1) .

Trait	Genotypes[a]			Effect size[b]	*P*
**chr16:70790626**	**CC**	**CT**			
Max N	1454	22		-	-
Sex (M/F)	645/809	9/13		1.006 (0.79, 1.29)	9.61×10^{-1}
Age (years)[c]	61.466 (19.27)	49.773 (19.80)		-0.706 (0.26)	6.00×10^{-3}
HDL (mmol/L)	1.267 (0.35)	0.779 (0.26)		-1.713 (0.25)	1.57×10^{-11}
LDL (mmol/L)	3.251 (0.95)	3.220 (1.17)		-0.088 (0.25)	7.31×10^{-1}
TC (mmol/L)	5.250 (1.09)	4.938 (1.24)		-0.364 (0.26)	1.54×10^{-1}
TG (mmol/L)	-0.042 (1.00)	0.543 (1.02)		0.577 (0.26)	2.62×10^{-2}
VLDL (mmol/L)	0.727 (0.43)	0.938 (0.46)		0.568 (0.26)	2.89×10^{-2}
**rs145556679**	**GG**	**GC**			
Max N	1438	38		-	-
Sex (M/F)	638/800	16/22		1.001 (0.85, 1.18)	9.92×10^{-1}
Age (years)[c]	61.393 (19.23)	57.043 (22.54)		-0.100 (0.17)	5.65×10^{-1}
HDL (mmol/L)	1.252 (0.35)	1.579 (0.40)		0.872 (0.17)	2.92×10^{-7}
LDL (mmol/L)	3.248 (0.95)	3.312 (1.04)		0.013 (0.17)	9.42×10^{-1}
TC (mmol/L)	5.244 (1.09)	5.307 (1.05)		0.021 (0.17)	$9.04 \times 10^{--1}$
TG (mmol/L)	-0.016 (0.99)	-1.255 (0.93)		-1.134 (0.17)	2.53×10^{-11}
VLDL (mmol/L)	0.738 (0.43)	0.416 (0.20)		-1.131 (0.17)	2.90×10^{-11}
**rs140087759**	**CC**	**CT**			
Max N	1445	30		-	-
Sex (M/F)	636/809	18/12		1.136 (0.94, 1.37)	1.76×10^{-1}
Age (years)[c]	61.399 (19.27)	55.222 (21.47)		0.162 (0.20)	4.12×10^{-1}
WHR (cm/cm)	0.926 (0.10)	1.008 (0.10)		-1.189 (0.21)	1.35×10^{-8}
**rs13382259**	**AA**	**TA**	**TT**		
Max N	1572	157	8	-	-
Sex (M/F)	495/1077	29/128	1/7	0.890 (0.84, 0.97)	3.95×10^{-3}
Age (years)[c]	45.018 (15.52)	45.116 (15.40)	37.625 (14.17)	0.010 (0.08)	9.01×10^{-1}
DBP (mm/Hg)	84.395 (12.56)	91.484 (15.71)	85.000 (12.91)	-0.554 (0.10)	3.18×10^{-8}
**rs6131100**	**TT**	**TA**	**AA**		
Max N	1603	133	1	-	-
Sex (M/F)	483/1120	42/91	0/1	1.00 (0.92, 1.07)	9.93×10^{-1}
Age (years)[c]	44.885 (15.43)	46.288 (16.33)	42	-0.046 (0.09)	6.13×10^{-1}
FGBMIadj (mmol/L)	4.843 (0.76)	4.341 (0.81)	-	0.790 (0.14)	1.21×10^{-8}
**rs79748197**	**AA**	**AG**	**GG**		
Max N	1710	26	1	-	-
Sex (M/F)	519/1191	6/20	0/1	0.967 (0.81, 1.15)	7.02×10^{-1}
Age (years)	44.951 (15.50)	47.656 (15.53)	-	-0.252 (0.21)	2.27×10^{-1}
WBC (10^9/L)	7.147 (1.71)	5.412 (1.11)	-	1.156 (0.21)	3.00×10^{-8}
**rs557129696**	**TT**	**TG**			
Max N	1723	14		-	-
Sex (M/F)	522/1201	3/11		1.159 (0.89, 1.51)	2.80×10^{-1}
Age (years)[c]	44.885 (15.51)	45.700 (14.77)		0.043 (0.30)	8.84×10^{-1}
HGB (g/dl)	13.875 (1.33)	11.169 (0.84)		-2.027 (0.31)	4.83×10^{-11}
**rs112037309**	**GG**	**AG**	**AA**		
Max N	2744	434	22	-	-
Sex (M/F)	1005/1739	166/268	6/16	0.100 (0.95, 1.04)	9.28×10^{-1}
Age (years)[c]	52.528 (19.22)	51.326 (18.61)	57.927 (20.69)	0.025 (0.05)	6.12×10^{-1}
Weight (kg)	74.441 (15.05)	79.635 (15.37)	75.465 (12.3)	-0.287 (0.05)	2.70×10^{-8}

Supplementary Table 6: Genotype summary statistics for the novel signals, including age and sex association results.

Max N, maximum sample number; HDL, high-density lipoprotein cholesterol; LDL, low-density lipoprotein cholesterol; TG, triglycerides; TC, total cholesterol; VLDL, very low density lipoprotein cholesterol; DBP, diastolic blood pressure; WHR, waist-to-hip ratio; FGBMIadj, fasting glucose adjusted for body mass index; WBC, white blood cells; HGB, haemoglobin; *P*, Wald test from the association analysis using METACARPA. [a]Untransformed mean (s.d.) except TG which is log transformed is presented as median (s.d.). [b]For continuous traits beta values (standard error) are reported, whereas for binary traits odds ratios and 95% confidence intervals are reported. [c]Inverse-normalised for analysis.

Population (code)	Allele 1	Allele 2
**Total 1000 Genomes dataset (ALL)**	**G:0.9998**	**C:0.0002**
**Total South Asian Ancestry (SAS)**	**G:1.0**	**C:0.0**
Bengali (BEB)	G:1.0	C:0.0
Gujarati Indian (GIH)	G:1.0	C:0.0
Indian Telugu (ITU)	G:1.0	C:0.0
Punjabi in Lahore (PJL)	G:1.0	C:0.0
Sri Lankan Tamil (STU)	G:1.0	C:0.0
Total East Asian Ancestry (EAS)	G:1.0	C:0.0
Chinese Dai in Xishuangbanna (CDX)	G:1.0	C:0.0
Han Chinese in Bejing (CHB)	G:1.0	C:0.0
Southern Han Chinese (CHS)	G:1.0	C:0.0
Japanese in Tokyo, Japan (JPT)	G:1.0	C:0.0
Kinh in Ho Chi Minh City (KHV)	G:1.0	C:0.0
Total Americas Ancestry (AMR)	G:1.0	C:0.0
Colombian (CLM)	G:1.0	C:0.0
Mexican Ancestry (MXL)	G:1.0	C:0.0
Peruvian (PEL)	G:1.0	C:0.0
Puerto Rican (PUR)	G:1.0	C:0.0
**Total African Ancestry (AFR)**	**G:1.0**	**C:0.0**
African Caribbean (ACB)	G:1.0	C:0.0
African American (ASW)	G:1.0	C:0.0
Esan (ESN)	G:1.0	C:0.0
Luhya (LWK)	G:1.0	C:0.0
Gambian (MAG)	G:1.0	C:0.0
Mende (MSL)	G:1.0	C:0.0
Yoruba (YRI)	G:1.0	C:0.0
**Total European Ancestry (EUR)**	**G:0.999**	**C:0.001**
Western European ancestry (CEU)	G:1.0	C:0.0
Finnish (FIN)	G:1.0	C:0.0
British (GBR)	G:1.0	C:0.0
Iberian (IBS)	G:1.0	C:0.0
Toscani (TSI)	G:0.9953	C:0.0047

Supplementary Table 7: Allele frequencies of rs145556679 in worldwide populations.

Phase 3 frequencies from the 1000 Genomes project.

Trait	Variant	Cohorts	Chr:pos	Reported variant	Reported variant chr:pos	Conditional *P*	Reported GWAS trait association	Reference PMID
HDL	Chr16:70790626	MANOLIS	16:70790626	NA				
TG	rs145556679	MANOLIS	11:117643264	rs2160669	11:116647607	3.40×10^{-11}	TG	24097068
				rs964184	11:116648917	1.04×10^{-10}	Lipid traits, coronary heart disease and hypertriglyceridemia	2068656, 24262325, 21378990, 24097068, 23505323, 20686565, 22003152, 22359512
				rs11823543	11:116649135	3.72×10^{-11}	TG-Blood Pressure	21386085
				rs12286037	11:116652207	3.72×10^{-11}	TG and lipoprotein-associated phospholipase A2 activity and mass	20442857, 18193043
				rs6589566	11:116652423	7.29×10^{-11}	LDL, TG	18179892, 23726366
				rs2075290	11:116653296	3.44×10^{-11}	HDL-TG	21386085
				rs603446	11:116654435	1.06×10^{-10}	TG	21909109
				rs2266788	11:116660686	7.51×10^{-11}	TG-Blood Pressure and HDL-TG	21386085
				rs651821	11:116662579	8.06×10^{-11}	Lipid traits & lipid metabolism phenotypes	22286219, 24386095, 22171074
				rs662799	11:116663707	8.02×10^{-11}	Lipid traits	24023260
				rs76353203	11:116701353	1.09×10^{-12}	HDL, TG	24343240
				rs2075292	11:116732512	3.22×10^{-11}	TG	18193046
				rs139961185[a]	11:116807343	1.72×10^{-10}	TG	24886709
				rs11216230	11:116884789	2.55×10^{-11}	HDL	24886709
				rs508487	11:117075566	4.73×10^{-11}	Cardiovascular disease risk factors	21943158
				rs10892151	11:117531731	2.29×10^{-11}	TG	19074352
				rs11603023	11:118486067	3.52×10^{-11}	TC	24097068
VLDL	rs145556679	MANOLIS	11:117643264	rs2160669	11:116647607	3.85×10^{-11}	TG	24097068
				rs964184	11:116648917	1.13×10^{-10}	Lipid traits, coronary heart disease and hypertriglyceridemia	2068656, 24262325, 21378990, 24097068, 23505323, 20686565, 22003152, 22359512
				rs11823543	11:116649135	4.21×10^{-11}	TG-Blood Pressure	21386085
				rs12286037	11:116652207	4.21×10^{-11}	TG and lipoprotein-associated phospholipase A2 activity and mass	20442857, 18193043
				rs6589566	11:116652423	8.05×10^{-11}	LDL, TG	18179892, 23726366
				rs2075290	11:116653296	3.89×10^{-11}	HDL-TG	21386085
				rs603446	11:116654435	9.71×10^{-10}	TG	21909109
				rs2266788	11:116660686	9.31×10^{-11}	TG-Blood Pressure and HDL-TG	21386085
				rs651821	11:116662579	8.95×10^{-10}	Lipid traits and lipid metabolism phenotypes	22286219, 24386095, 22171074
				rs662799	11:116663707	8.91×10^{-11}	Lipid traits	24023260
				rs76353203	11:116701353	1.22×10^{-12}	HDL, TG	24343240
				rs2075292	11:116732512	3.63×10^{-11}	TG	18193046
				rs139961185[a]	11:116807343	2.31×10^{-10}	TG	24886709
				rs11216230	11:116884789	3.00×10^{-11}	HDL	24886709
				rs508487	11:117075566	5.77×10^{-11}	Cardiovascular disease risk factors	21943158
				rs10892151	11:117531731	2.61×10^{-11}	TG	19074352
				rs11603023	11:118486067	3.93×10^{-11}	TC	24097068
DBP	rs816463	MANOLIS	5:4872780	NA				
WHR	rs140087759	MANOLIS	5:28292892	NA				
DBP	rs13382259	Pomak	2:113934176	NA				
FGBMIadj	rs6131100	Pomak	20:10434530	NA				
WBC	rs79748197	Pomak	2:19430105	NA				
HGB	rs557129696[b]	Pomak	11:5328683	rs7116019	11:4618606	5.19×10^{-11}	MCV, MCHC, MCH	25373335
				rs12788102	11:4790575	4.47×10^{-11}	Malaria	23717212
				rs12274659	11:4947444	5.28×10^{-11}	MCV	25373335
				rs11035019	11:5009563	5.28×10^{-11}	MCV	25373335
				rs2445284	11:5029703	7.04×10^{-11}	Sickle cell anemia (haemolysis)	23406172
				rs7950726	11:5225447	3.59×10^{-10}	HbA_2 levels	23043469
				rs11036238	11:5225635	6.73×10^{-11}	Malaria	19465909
				rs2071348	11:5264146	6.37×10^{-11}	Beta thalassemia/haemoglobin E disease	20183929
				rs4910742	11:5306509	5.08×10^{-11}	Foetal haemoglobin levels and Inflammatory biomarkers	18245381
				rs5006884	11:5373251	4.82×10^{-11}	Foetal haemoglobin levels	20018918
				rs7948471	11:5471746	7.24×10^{-11}	Sickle cell anemia (haemolysis)	23406172
				rs37209[c]	11:5518156	1.22×10^{-8}	Malaria	22895189
Weight	rs112037309	MANOLIS and Pomak	4:106617136	NA				

Supplementary Table 8: Conditional analysis. Conditional analysis results for the novel variants using previously reported genome-wide significant (P ≤ 5.00 x 10-8) variants within 1Mb.

Cohorts, cohorts in which the signal arose; Chr:position represents the chromosome and position in GRCh37/hg19 coordinates; Reported variant, RS-id of the reported signal variant, Reported variant chr:pos, the chromosome and position in GRCh37/hg19 coordinatesfor the reported variant; Conditional *P* , this is the Wald test from METACARPA after conditioning on the reported variant; Reference PMID, reference for the known GWAS signal. The abbreviations are: TG, triglycerides; VLDL, very low density lipoprotein cholesterol; HDL, high-density lipoprotein cholesterol; DBP, diastolic blood pressure; WHR, waist-to-hip ratio; FGBMIadj, fasting glucose adjusted for body mass index; WBC, white blood cells; HGB, haemoglobin; LDL, low-density lipoprotein cholesterol; TC, total cholesterol; MCV, mean corpuscular volume; MCHC, mean corpuscular haemoglobin concentration; MCH, mean corpuscular haemoglobin; HbA_2, haemoglobin A_2. [a] rs139961185 was only present in MANOLIS OmniExome therefore the conditional *P* is the likelihood ratio test from the association analysis using GEMMA. [b] rs7120391 is a known GWAS hit for RBC trait and rs4910742 (chr11:5306509) is a known GWAS hit for foetal haemoglobin levels these variants were not present in the Pomak data and therefore conditional analysis could not be performed. [c] rs37209 was only present in Pomak OmniExome therefore the conditional *P* is the likelihood ratio test from the association analysis using GEMMA.

	Inside LCR (Inaccessible)			*Outside LCR (accessible)*		
	**Raw**	**VQSR-filtered**	**Phased**	**Raw**	**VQSR-filtered**	**Phased**
**Number of sites**	2049810	491003	152287	34363557	10315908	9411321
**Total length**	297790473			2842254171		
**Fraction of genome**	9.48%			90.5%		
**SNP Density (SNP/Kbp)**	6.88	1.65	0.51	12.09	3.63	3.31
**Number (Fraction) of rare (MAF<1%) sites**	482955 (28.7%)	55909 (18.3%)	18984 (12.5%)	25475182 (69.5%)	3012997 (29.5%)	1661168 (17.65%)
**Number (Fraction) of low-frequency (1%<MAF<5%) sites**	573190 (34%)	44917 (14.7%)	39884 (26.2%)	4605358 (12.6%)	2324450 (22.7%)	2301572 (24.4%)
**Number (Fraction) of common (5%<MAF) sites**	622851 (37%)	204498 (66.9%)	93419 (61.3%)	6581113 (17.9%)	4884646 (47.8%)	5448581 (57.9%)

Supplementary Table 9: Variant densities in low complexity regions.

Consequence group	Consequence term
3'/5' UTR	3 prime UTR variant,<br>5 prime UTR variant
Coding sequence variant	coding sequence variant,<br>incomplete terminal codon variant,<br>initiator codon variant,<br>missense variant,<br>start lost,<br>stop gained,<br>stop lost,<br>stop retained variant,<br>synonymous variant
Intergenic variant	intergenic variant,
Regulatory variant	regulatory region variant,<br>TF binding site variant
Intron variant	intron variant
Splice-region variant	splice acceptor variant,<br>splice donor variant,<br>splice region variant
Upstream/Downstream gene variant	downstream gene variant,<br>upstream gene variant
Non-coding transcript variant	mature miRNA variant,<br>Non coding transcript variant,<br>non coding exon variant,<br>non coding transcript exon variant

Supplementary Table 10: MANOLIS sequence variant consequence grouping.

Trait	Derived information	Unit of measurement	Exclusions	Filter	Covariates used
BMI	Weight/height^2	kg/m^2		> 4SDs	Age,age^2
CRP		mg/L	< 0.1mg/L or > 10mg/L	< or >3SDs	Age,age^2
DBP[§]		mmHg	Age >70 yrs	< or > 5SDs	Age,age^2,BMI
FG		mmol/L	> 7mmol/L		Age,age^2
FGBMIadj		mmol/L	> 7mmol/L		Age,age^2,BMI
FI		μIU/ml		< or > 5SDs	Age,age^2
FIBMIadj		μIU/ml		< or > 5SDs	Age,age^2,BMI
Height		cm		> 4SDs	Age,age^2
HGB		g/dl		< or > 3SDs	Age,age^2
HDL		mmol/L		< or > 5SDs	
Hip		cm		> 4SDs	Age,age^2
HipBMIadj		cm		> 4SDs	Age,age^2,BMI
HOMA_ir	(FI (μIU/ml) × FG (mmol/L))/22.5			< or > 5SDs	Age,age^2
HOMA_irBMIadj	(FI (μIU/ml) × FG (mmol/L))/22.5			< or > 5SDs	Age,age^2,BMI
LDL		mmol/L		< or > 5SDs	
MCH		pg		< or >3SDs	Age,age^2
MCHC		g/dL		< or >3SDs	Age,age^2
MCV		fl		< or >3SDs	Age,age^2
PCV		%		< or >3SDs	Age,age^2
PLT		10^9/L		< or >3SDs	Age,age^2
RBC		10^{12}/L		< or > 3SDs	
SBP[§]		mmHg	Age > 70 yrs	< or > 5SDs	Age,age^2,BMI
TC		mmol/L		< or > 5SDs	
TG		mmol/L		< or > 5SDs	
VLDL	TC-HDL-LDL	mmol/L			
Waist		cm		> 4SDs	Age,age^2
WaistBMIadj		cm		> 4SDs	Age,age^2,BMI
WBC		10^9/L		< or >3SDs	Age,age^2
Weight		kg		> 4SDs	Age,age^2
WHR	Waist/hip	cm/cm		> 4SDs	Age,age^2
WHRBMIadj	Waist/hip	cm/cm		> 4SDs	Age,age^2,BMI

Supplementary Table 11: Phenotype preparation.

The phenotypes were prepared separately for the HELIC cohort arrays. If the trait is derived the information for this is contained in the column headed derived information. [§]Individuals known to be taking anti-hypertensive or blood pressure lowering medication had their measurement adjusted by adding 15mmHg to raw SBP and 10mmHg to raw DBP. The trait abbreviations are: BMI, body mass index; CRP, C-reactive protein; DBP, diastolic blood pressure; FG, fasting glucose; FGBMIadj, fasting glucose adjusted for BMI; FI, fasting insulin; FIBMIadj, fasting insulin adjusted for BMI; HGB, haemoglobin; HDL, high-density lipoprotein cholesterol; HipBMIadj, hip adjusted for BMI; HOMA_ir, homeostasis model assessment of insulin resistance; HOMA_irBMIadj, homeostasis model assessment of insulin resistance adjusted for BMI; LDL, low-density lipoprotein cholesterol; MCH, mean corpuscular haemoglobin; MCHC, mean corpuscular haemoglobin concentration; MCV, mean corpuscular volume; PCV, haematocrit; PLT, platelets; RBC, red blood cells; SBP, systolic blood pressure; TC, total cholesterol; TG, triglycerides; VLDL, very low density lipoprotein cholesterol; WBC, white blood cells; WaistBMIadj, waist adjusted for BMI; WHR, waist-to-hip ratio; WHRBMIadj, waist hip ratio adjusted for BMI. All traits were inverse normal transformed except for WBC and TG which were log transformed and MCHC was untransformed.

Cohort	HELIC MANOLIS OmniExome				HELIC MANOLIS CoreExome				HELIC Pomak OmniExome				HELIC Pomak CoreExome			
N	1265				211				1003				734			
% females	57.31				45.97				72.48				66.08			
Mean age (range)	62.06 (18 - 98)				56.7 (18 - 91)				43.3 (16 - 92)				47.21 (18.2 - 89.8)			
Trait	N	Mean (sd)	Range (min - max)	Lambda	N	Mean (sd)	Range (min - max)	Lambda	N	Mean (sd)	Range (min - max)	Lambda	N	Mean (sd)	Range (min - max)	Lambda
BMI	1020	29.47 (5.16)	16.98 - 50.11	1.003	204	29.76 (4.9)	19.44 - 44	1.001	935	27.8 (5.35)	16 - 47.47	0.992	725	27.34 (5.59)	15.43 - 46.49	1.001
CRP	1068	2.35 (1.9)	0.3 - 8.7	1.023	188	2.32 (1.76)	0.3 - 8.1	1.205	826	1.81 (1.64)	0.3 - 7.7	0.989	607	1.75 (1.54)	0.3 - 7	1.034
DBP	574	82.46 (12.13)	52 - 123	1.01	115	81.43 (12.71)	51 - 128	1.027	551	87.57 (13.29)	50 - 133	0.998	641	82.72 (12.27)	60 - 120	1.008
FG	701	5.2 (0.78)	1.72 - 6.99	0.995	31	4.29 (0.56)	3.22 - 5.66	1.113	177	5.17 (0.75)	2.5 - 6.99	0.976	574	4.69 (0.74)	2.05 - 6.99	1.022
FGBMIadj	610	5.24 (0.77)	1.72 - 6.99	1.001	31	4.29 (0.56)	3.22 - 5.66	1.077	174	5.18 (0.75)	2.5 - 6.99	0.971	569	4.69 (0.75)	2.05 - 6.99	1.025
FI	789	12.11 (9.96)	2 - 71	1.026	35	12.69 (7.23)	4 - 36	1.05	196	12.03 (7.75)	2 - 49	1.001	618	14.63 (13.49)	2 - 74	1.033
FIBMIadj	690	12.24 (10.33)	2 - 75	1.003	34	12.41 (7.16)	4 - 36	0.986	193	12.07 (7.81)	2 - 49	0.985	613	14.69 (13.52)	2 - 74	1.038
HDL	1255	1.28 (0.36)	0.41 - 2.54	0.991	210	1.15 (0.33)	0.41 - 2.15	1.143	992	1.15 (0.31)	0.36 - 2.28	0.998	715	1.22 (0.33)	0.47 - 2.51	1.008
Height	1044	162.45 (9.96)	135 - 192	0.983	204	163.3 (10.26)	139 - 186	0.979	936	162.31 (9.62)	120 - 196	1.028	732	163.55 (10.01)	138 - 199	1.006
HGB	992	14.1 (1.49)	9.9 - 18.2	0.992	205	14.13 (1.56)	9.7 - 17.9	1.032	945	13.8 (1.37)	9.7 - 17.9	1.01	717	13.93 (1.31)	10 - 18.2	0.994
Hip	1048	107.86 (9.95)	73.3 - 148	1.008	204	105.93 (9.34)	85 - 130.5	0.993	880	105.81 (10.54)	74 - 150	1	727	106.07 (10.45)	78 - 153	1.009
HipBMIadj	996	107.77 (9.86)	73.3 - 145	0.99	203	105.88 (9.33)	85 - 130.5	0.962	868	105.72 (10.49)	74 - 150	0.985	724	105.94 (10.2)	78 - 142	1.005
HOMA_ir	792	3.25 (3.26)	0.34 - 22.53	1.01	35	3.04 (2.94)	0.71 - 13.59	1.079	194	3.08 (2.65)	0.47 - 18.25	1.109	615	3.33 (3.59)	0.35 - 22.79	1.028
HOMA_irBMIadj	693	3.33 (3.42)	0.34 - 23.43	1.003	34	2.91 (2.89)	0.71 - 13.59	1.022	191	3.1 (2.67)	0.47 - 18.25	0.996	610	3.35 (3.6)	0.35 - 22.79	1.031
LDL	1252	3.27 (0.95)	0.54 - 8.08	1.001	210	3.12 (0.96)	0.85 - 6.66	1.002	991	3.15 (0.93)	0.67 - 7.1	0.984	715	3.02 (0.93)	0.8 - 6.4	1.005
MCH	981	29.1 (2.38)	20.9 - 34.8	1.009	201	29.52 (2.32)	21.1 - 35.1	0.976	935	28.96 (1.9)	21.3 - 35.3	0.978	712	29.41 (1.85)	22 - 35.5	1.006
MCHC	994	34.37 (0.85)	31.9 - 36.8	1.011	206	33.88 (1.17)	30.9 - 36.5	1.053	947	33.16 (1.1)	29.6 - 36.6	0.978	722	33.47 (1.15)	29.6 - 37.2	0.98
MCV	978	84.64 (6.34)	61.5 - 98.5	1.002	202	86.89 (5.98)	65.5 - 102.3	0.971	939	87.1 (5.07)	68.4 - 101.8	0.971	719	87.53 (4.81)	70.6 - 102.9	0.992
PCV	994	41.02 (4.23)	29.1 - 53.3	0.99	204	41.77 (4.36)	30.2 - 54.3	1.054	947	41.58 (3.6)	31.3 - 52.7	1.024	726	41.45 (3.5)	31.3 - 51	1.007
PLT	992	211.31 (50.33)	46 - 384	1.004	203	205.64 (49.45)	65 - 359	1.018	951	242.06 (54.85)	94 - 419	0.986	725	238.54 (55.39)	97 - 415	1.005
RBC	1015	4.89 (0.55)	3.27 - 6.83	1.017	208	4.85 (0.58)	3.3 - 6.85	1.027	992	4.78 (0.42)	3.62 - 6.39	1.002	726	4.75 (0.41)	3.57 - 5.88	1.024
SBP	575	137.5 (22.15)	89 - 217	0.999	115	136.75 (20.66)	99 - 222	1.057	551	138.77 (23.07)	94 - 232	0.995	641	129.93 (18.23)	100 - 185	1.003
TC	1254	5.29 (1.09)	2.23 - 10.59	0.998	210	5 (1.06)	2.69 - 8.47	1.003	991	5.01 (1.05)	2.25 - 9.4	0.99	715	5.01 (1.04)	2.33 - 8.86	1.004
TG	1252	1.59 (0.93)	0.35 - 8.05	0.995	209	1.57 (0.87)	0.28 - 6.14	0.989	989	1.53 (0.83)	0.33 - 6.2	1.011	714	1.69 (0.9)	0.41 - 6.27	1.024
VLDL	1253	0.73 (0.43)	0.16 - 3.68	0.997	210	0.73 (0.44)	0.13 - 3.34	0.995	992	0.71 (0.4)	0.16 - 3.37	1.002	715	0.78 (0.44)	0.18 - 3.26	1.009
Waist	1057	98.88 (13.44)	62 - 141	1.008	204	104.75 (13)	68.5 - 142	1.003	889	90.34 (14.55)	58 - 147	0.993	729	95.09 (16.13)	58 - 152	1.009
WaistBMIadj	1002	98.6 (13.45)	62 - 137	0.985	203	104.76 (13.04)	68.5 - 142	0.988	877	90.25 (14.47)	58 - 147	0.995	726	95.06 (16.1)	58 - 152	0.996
WBC	986	6.96 (1.7)	2.6 - 12.5	1.01	203	6.99 (1.78)	2.15 - 11.5	0.995	948	6.98 (1.72)	2.6 - 12.2	1.018	725	7.3 (1.69)	2.9 - 12.6	1.026
Weight	1053	77.63 (15.23)	38.7 - 143	1.003	205	79.59 (15.75)	42 - 130	1.021	945	73.17 (14.55)	36 - 126	1.009	727	72.98 (15)	39 - 131	1
WHR	1047	0.92 (0.1)	0.63 - 1.19	1	204	0.99 (0.09)	0.72 - 1.2	1.016	874	0.85 (0.1)	0.65 - 1.13	1.008	727	0.89 (0.1)	0.62 - 1.18	1.001
WHRBMIadj	995	0.91 (0.1)	0.63 - 1.18	0.995	203	0.99 (0.09)	0.72 - 1.2	1.01	862	0.85 (0.1)	0.65 - 1.13	1.004	725	0.89 (0.1)	0.62 - 1.18	1.007

Supplementary Table 12: Phenotype summary statistics. The summary statistics for the raw data for each cohort set.
The abbreviations are: N, number of samples; BMI, body mass index; CRP, C-reactive protein; DBP, diastolic blood pressure; FG, fasting glucose; FGBMIadj, fasting glucose adjusted for BMI; FI, fasting insulin; FIBMIadj, fasting insulin adjusted for BMI; HGB, haemoglobin; HDL, high-density lipoprotein cholesterol; HipBMIadj, hip adjusted for BMI; HOMA_ir, homeostasis model assessment of insulin resistance; HOMA_irBMIadj, homeostasis model assessment of insulin resistance adjusted for BMI; LDL, low-density lipoprotein cholesterol; MCH, mean corpuscular haemoglobin; MCHC, mean corpuscular haemoglobin concentration; MCV, mean corpuscular volume; PCV, haematocrit; PLT, platelets; RBC, red blood cells; SBP, systolic blood pressure; TC, total cholesterol; TG, triglycerides; VLDL, very low density lipoprotein cholesterol; WBC, white blood cells; WaistBMIadj, waist adjusted for BMI; WHR, waist-to-hip ratio; WHRBMIadj, waist-to-hip ratio adjusted for BMI.

Supplementary Note 1: Replicated known signals

For rs7412, which has been shown to be associated with lipid traits in Finnish populations[1], we find an association with both low-density lipoprotein (LDL) levels (beta -0.42 (SE 0.047), P = 2.64 x 10^{-19}, EAF = 0.079) and total cholesterol (TC) levels (beta -0.27 (SE 0.047), P = 1.05 x 10^{-8}, EAF = 0.079) (Table 1). Rs7412 is a missense variant located in *APOE.*

We also replicate a previously reported[2] association across both populations with C-reactive protein (CRP) and rs7553007 (beta -0.20 (SE 0.03), P = 6.80 x 10^{-12}, EAF = 0.327) (Table 1).

rs964184, another previously published[3] directly typed common variant, was associated in both populations with triglyceride levels (TG) (beta 0.24 (SE 0.03), P = 1.52x10^{-11}, EAF = 0.163) (Table 1) and very low-density lipoprotein levels (VLDL) (beta 0.24 (SE 0.03), P = 3.68x10^{-12}, EAF = 0.163) (Table 1).

Rs76353203 is directly typed and associated with HDL (beta 0.92 (SE 0.13), P = 1.78 x 10^{-12}, EAF = 0.022) and TG (beta -1.07 (SE 0.13), P = 6.88 x 10^{-17}, EAF = 0.022) (Table 1) in the MANOLIS cohort. These new data confirmed the association of rs76353203 with HDL and TG that were previously associated in 1267 samples genotyped on the exome chip in MANOLIS[4].

We performed conditional analysis for those variants located within 1Mb of a known GWAS hit for the same trait and replicated 5 additional signals.

In both cohorts we observe associations with the indel rs150641967 and lipids: LDL (beta -0.33 (SE 0.05), P = 3.49 x 10^{-11}, EAF = 0.075), TC (beta -0.32 (SE 0.05), P = 8.29 x 10^{-11}, EAF = 0.074), TG (beta -0.28 (SE 0.05), P = 2.49 x 10^{-8}, EAF=0.074) and VLDL (beta -0.28 (SE 0.05), P = 1.48 x 10^{-8}, EAF = 0.075) (Table 1). rs150641967 resides in an intron of *HAPLN4* and conditional analysis revealed this was the same signal as rs10401969 located in an intron of *SUGP1* associated with LDL, TG and TC[3].

We found an association between HDL in both populations and rs35237252 (beta 0.18 (SE 0.03), P = 4.04 x 10^{-10}, EAF = 0.277) (Table 1), a regulatory region variant that overlaps with a promoter flanking region, which was found to be active in skeletal muscle and fibroblast cells[5]. rs35237252 was conditionally dependent on rs2083637, a previously identified intergenic variant[6] (Table 1).

A further association in both populations was identified for HDL at rs200751500 (beta 0.29 (SE 0.03), P = 4.02 x 10^{-25}, EAF=0.330). The variant is situated in an intron of the cholesteryl ester transfer protein, plasma (*CETP*) gene, which also overlaps with a promoter flanking region found to be active in most tissues[5]. rs200751500 is conditionally dependent on another *CETP* intron variant rs1532624 previously reported for HDL in a large study comprising 17k samples from 16 different European regions[6].

We observed an association between mean corpuscular haemoglobin (MCH) in both populations (beta 0.20 (SE 0.03), $P = 1.90 \times 10^{-9}$ EAF = 0.228) (Table 1) and rs1331309. This signal could not be distinguished from numerous known blood cell related trait associations in this region, for example rs7775698, which is associated with MCH in Europeans[7].

Finally, rs9804550 was found to be associated with white blood cells (WBC) in the Pomak population (beta 0.52 (SE 0.08), $P = 1.10 \times 10^{-10}$, EAF = 0.051) (Table 1). This was attenuated when taking into account a signal that we have previously reported for rs7116019 for blood traits in the same cohort[8]. This signal is also seen in other blood traits in the Pomak population: mean corpuscular haemoglobin (MCH) (beta -0.63 (SE 0.08), $P = 2.19 \times 10^{-15}$, EAF = 0.053) (Table 1); mean corpuscular haemoglobin concentration (MCHC), (beta 0.89 (SE 0.075), $P = 8.46 \times 10^{-33}$, EAF = 0.054) (Table 1); mean corpuscular volume (MCV) (beta -1.07 (SE 0.08), $P = 1.57 \times 10^{-45}$, EAF = 0.052) (Table 1); red blood cells (RBC (beta 0.47 (SE 0.08), $P = 8.58 \times 10^{-10}$ EAF = 0.054) (Table 1).

Supplementary Note 2: Low-complexity regions

In the low complexity regions reported by Li, H *et al*[9], which cover about 10% of the GRCh37 build, SNP density is much lower compared to the rest of the genome, at 6.8 SNPs/Kbp vs. 12.09 SNPs/Kbp (Supplementary Table 9). Filtering further reduces this ratio; in the phased dataset there are 6.5 times more SNPs per kilobase in the mappable genome than there are in the LCR. Although the allelic makeup of the variants in these regions is different from the average, we do not observe an excess of rare variants in the LCRs.

Supplementary Note 3: Genotype concordance between HELIC MANOLIS WGS and imputed data.

There are 243 MANOLIS samples that overlap between the sequenced samples, included in the reference panel, and the imputed samples. We compared the imputed genotypes to the sequenced ones and find that the minor allele concordance is on average 94.2% for rare variants (<1%), 97.4% for low-frequency variants (1%<MAF<5%), and 99.7% for common variants (MAF>5%).

Supplementary Methods

Fine-mapping

We applied a method that assigns a relative probability of regulatory function (PRF) score among candidate causal variants, reweighting association statistics based on epigenomic annotations. Briefly, we collected a set of 70 genomic and epigenomic annotations, primarily GENCODE gene annotations (v19), FANTOM transcription start sites and enhancers[10,11], and imputed Roadmap Epigenomics histone marks, DNase hypersensitivity, and ChromHMM genome segmentations for the lymphoblastoid cell line epigenome (GM12878)[12,13]. We used the fgwas software[14] to train a Bayesian hierarchical model to compute enrichment of eQTLs in these annotations based on summary statistics from the Geuvadis RNA- sequencing

project[15]. We used forward stepwise selection followed by cross-validation to arrive at a combined model with 37 annotations and their associated enrichments. The respective annotations from 119 Roadmap epigenomes were used to compute PRF scores in each of the 119 epigenomes. At each locus we selected the top four epigenomes based on the maximum regulatory score among variants in the 95% credible set, and examined the regulatory annotations for variants in the credible set. We attempted to fine-map all novel signals using a window of 1Mb either side of the index variant.

Array genotyping and quality control (QC)

HumanExome

PLINK[16] was used to carry out the QC steps and variants were mapped to NCBI build 37 (hg19) coordinates and strand was standardised (http://www.well.ox.ac.uk/~wrayner/strand/). Post-Gencall all samples and variants with a call rate < 90% were excluded. Samples that displayed a call rate lower than 98%, sex discrepancies, or that violated identity to the OmniExpress genotypes were excluded, as well as heterozygosity outliers visualised using 2 different MAF bins (< 1% MAF and ≥ 1% MAF). Duplicates were excluded on the basis of identity by descent (IBD) calculated on low-frequency and common (MAF > 1%), LD pruned (r^2 < 0.2) variants not located in complex regions. In any given pair that displayed $\hat{\pi} > 0.9$, we rejected the duplicate with the lowest call rate. Ethnic outliers were identified and excluded by merging with 1000 Genomes Project data[17] and carrying out IBD (as described above) followed by multi-dimensional scaling (MDS). zCall calling was performed using only those samples passing QC. Post zCall variants were excluded with GenCall call rate < 95%, a GenCall cluster separation score < 0.4, a zCall call rate < 99% and HWE $P < 1 \times 10^{-4}$. A total of 1267 MANOLIS samples with 239,606 variants and 1012 Pomak samples with 239,596 variants passed QC. The genotypes from the OmniExpress and HumanExome chips were merged into a single dataset.

HumanCoreExome

QC was performed as described above, except for the following changes. Identity checks were performed by comparing to Sequenom MassARRAY® genotypes, obtained at sample reception, to the array genotypes. For the duplicate identification and ethnicity QC, additional IBD and MDS analyses were carried out, in which the CoreExome samples were combined with the OmniExome samples.

Validation assay

Assays for all SNPs were designed using the eXTEND suite and MassARRAY Assay Design software version 4.0.0.2 (Agena Bioscience, Inc.). Amplification was performed in a total volume of 5µL containing ~10ng genomic DNA, 100nM of each PCR primer, 500µM of each dNTP, 1.25 x PCR buffer (Qiagen), 1.625mM $MgCl_2$ and 1U HotStar Taq® (Qiagen). Reactions were heated to 94 °C for 15 min followed by 45 cycles at 94 °C for 20 s, 56 °C for 30 s and 72 °C for 1 min, then a final extension at 72 °C for 3 min. Unincorporated dNTPs were SAP digested prior to iPLEX™ allele specific extension with mass-modified ddNTPs using an iPLEX reagent kit (Agena Bioscience, Inc.). SAP digestion and extension were performed according to the manufacturer’s instructions with reaction extension primer concentrations adjusted to 0.7-1.8µM,

dependent upon primer mass. Extension products were desalted and dispensed onto a SpectroCHIP using a MassARRAY Nanodispenser prior to MALDI-TOF analysis with a MassARRAY Analyzer Compact mass spectrometer. Genotypes were automatically assigned and manually confirmed using MassARRAY TyperAnalyzer software version 4.0 (Agena Bioscience, Inc.). QC was carried out separately for each iPLEX. Samples with call rate < 80% were excluded. Variants with call rate < 90% or HWE $P < 1 \times 10^{-4}$ were excluded. To test the concordance of imputed and directly typed genotypes, genotype dosages were converted to genotype calls, those with an uncertainty greater than 0.4 were set to missing and the rest were treated as hard calls. If the prioritised variant was already directly typed we visualised the array intensities using Scattershot (http://www.well.ox.ac.uk/~wrayner/tools/).

References

1 Kettunen, J. *et al.* Genome-wide association study identifies multiple loci influencing human serum metabolite levels. *Nature genetics* **44**, 269-276, doi:10.1038/ng.1073 (2012).

2 Elliott, P. *et al.* Genetic Loci associated with C-reactive protein levels and risk of coronary heart disease. *Jama* **302**, 37-48, doi:10.1001/jama.2009.954 (2009).

3 Willer, C. J. *et al.* Discovery and refinement of loci associated with lipid levels. *Nature genetics* **45**, 1274-1283, doi:10.1038/ng.2797 (2013).

4 Tachmazidou, I. *et al.* A rare functional cardioprotective APOC3 variant has risen in frequency in distinct population isolates. *Nature communications* **4**, 2872, doi:10.1038/ncomms3872 (2013).

5 Flicek, P. *et al.* Ensembl 2014. *Nucleic acids research* **42**, D749-755, doi:10.1093/nar/gkt1196 (2014).

6 Aulchenko, Y. S. *et al.* Loci influencing lipid levels and coronary heart disease risk in 16 European population cohorts. *Nature genetics* **41**, 47-55, doi:10.1038/ng.269 (2009).

7 Ferreira, M. A. *et al.* Sequence variants in three loci influence monocyte counts and erythrocyte volume. *American journal of human genetics* **85**, 745-749, doi:10.1016/j.ajhg.2009.10.005 (2009).

8 Panoutsopoulou, K. *et al.* Genetic characterization of Greek population isolates reveals strong genetic drift at missense and trait-associated variants. *Nature communications* **5**, 5345, doi:10.1038/ncomms6345 (2014).

9 Li, H. Toward better understanding of artifacts in variant calling from high-coverage samples. *Bioinformatics* **30**, 2843-2851, doi:10.1093/bioinformatics/btu356 (2014).

10 Forrest, A. R. *et al.* A promoter-level mammalian expression atlas. *Nature* **507**, 462-470, doi:10.1038/nature13182 (2014).

11 Andersson, R. *et al.* An atlas of active enhancers across human cell types and tissues. *Nature* **507**, 455-461, doi:10.1038/nature12787 (2014).

12 Ernst, J. & Kellis, M. Large-scale imputation of epigenomic datasets for systematic annotation of diverse human tissues. *Nature biotechnology* **33**, 364-376, doi:10.1038/nbt.3157 (2015).

13 Kundaje, A. *et al.* Integrative analysis of 111 reference human epigenomes. *Nature* **518**, 317-330, doi:10.1038/nature14248 (2015).

14 Pickrell, J. K. Joint analysis of functional genomic data and genome-wide association studies of 18 human traits. *American journal of human genetics* **94**, 559-573, doi:10.1016/j.ajhg.2014.03.004 (2014).
